# Racial disparities in breast cancer subtypes, recurrence, and survival: a longitudinal cohort analysis

**DOI:** 10.3389/fonc.2026.1732495

**Published:** 2026-03-25

**Authors:** Rami Addasi, Basma AbuMahfouz, Aseel Subuh, Maysam Obeidat, Adam Zenah, Rahaf Haddar, Samar Hamdan

**Affiliations:** 1 Department of General Surgery, School of Medicine, The University of Jordan, Amman, Jordan; 2 Department of Undergraduate Studies, School of Medicine, The University of Jordan, Amman, Jordan; 3 Department of Internal Medicine, School of Medicine, The University of Jordan, Amman, Jordan

**Keywords:** breast cancer, invasive ductal carcinoma, invasive lobular carcinoma, racial disparities, recurrence, survival analysis, triple-negative breast cancer

## Abstract

**Introduction:**

Racial disparities in breast cancer subtype distribution and clinical outcomes are well documented, yet integrated longitudinal analyses examining subtype, recurrence, and survival within standardized cohorts remain limited. This study examines race-associated differences in breast cancer subtypes, recurrence patterns, and survival outcomes using a retrospective longitudinal cohort.

**Methods:**

A total of 922 women from the Duke Breast Cancer MRI dataset were analyzed. Race was categorized *a priori* into three groups (White, Black, and Other) for primary comparative analyses.

**Results:**

The median age at diagnosis (in years) was 52.2 (IQR 45.4-60.8), with Black patients presenting at younger median ages compared with White patients (p< 0.001). Black women had the highest prevalence of triple-negative breast cancer (29.6%). Sixty-five (47.1%) of black patients presented with stage III Nottingham grade at presentation (P< 0.001). The overall recurrence rate was 9.4%, and mortality rate was 6.7%, with no statistically significant difference between groups.

**Discussion:**

Black patients demonstrated a higher prevalence of aggressive tumor biology at presentation; however, survival differences were attenuated after multivariable adjustment. Given the limited number of mortality events, adjusted survival estimates should be interpreted cautiously, as these findings underscore the need for larger, prospective studies integrating genomic, imaging, and socioeconomic data to better define drivers of outcome disparities in breast cancer.

## 1 Introduction

Breast cancer remains a leading cause of cancer-related morbidity and mortality among females worldwide, despite advances in screening technology that have improved the accuracy of detection, diagnosis and treatment (1). In 2020 alone, around 2.3 million new cases of breast cancer were diagnosed, with nearly 685,000 death reported globally (2). Despite ongoing advancements in diagnosis and treatment, persistent racial disparities in breast cancer incidence, tumor biology, prognosis, and survival continue to pose significant challenges to achieving equitable healthcare (3). For instance, women of African descent are often diagnosed at later stages resulting in a higher mortality rate compared with White women, even when diagnosed at similar stages of the disease (4). These disparities are attributed to multiple factors, including socioeconomic status, environmental exposure, access to health care and a higher prevalence of aggressive tumor subtypes, such as triple-negative breast cancer (TNBC) among women of African descent (3–6). Although many studies have reported racial differences in breast cancer outcomes, few have used long−term cohorts with consistent subtype classification, recurrence data, and survival follow−up to examine disparities across multiple stages of the disease.

Racial disparities are a well‐documented challenge in medicine, particularly in cancer care. Studies have shown that racial and ethnic minority groups often experience delayed diagnoses, higher rates of late−stage presentation, and poorer survival compared with White patients. Moreover, population-specific aggregation of pathogenic variants such as BRCA1/2 and TP53 has been observed across multiple cancer types, including breast, ovarian, prostate, and pancreatic malignancies, with direct implications for targeted therapies (7). These patterns highlight the clinical importance of understanding race-associated tumor biology within outcome-focused cohorts. Although genetic variation can influence cancers’ susceptibility and tumor biology, it explains only a small portion of observed disparities. For example, Black cancer patients report direct experiences of racial discrimination that influence care quality and contribute to disparities in medical care (8). Large cohort studies also demonstrate that Asian, Black, and Native Hawaiian or Other Pacific Islander adolescents and young adults face significantly higher risks of late−stage cancer diagnosis and reduced survival relative to White peers (9). Together, these findings underscore the need for more equity−focused research to comprehensively address racial disparities in cancer.

Previous research consistently demonstrated racial differences in breast cancer outcomes. However, relatively few studies combine imaging racial disparities in breast cancer outcomes, using few high-resolution imaging datasets alongside clinical markers in longitudinal cohorts to correlate the tumor characteristics with prognosis across racial groups (10). Moreover, most existing work focuses on either population-level epidemiology or isolated imaging endpoints rather than integrating subtype and outcome data within the same patients. These gaps highlight the need for research that evaluate recurrence and survival patterns within diagnostically standardized cohorts, in addition to investigating clinical differences across racial groups (11).

Breast magnetic resonance imaging (MRI) plays an established role in the evaluation of tumor extent and treatment planning in patients with breast cancer, and emerging studies have explored the prognostic potential of MRI-derived imaging (12). Black women are significantly less likely to receive preoperative breast MRI compared to White women. Ginzberg et al. (13) found that Black patients had lower odds of undergoing preoperative MRI in a cohort of 1,410 women with stage 0-III breast cancer. Black women who received MRI were less likely to require re-excision surgery compared to those who did not. This suggests that standardizing MRI use and understanding the different pathological presentations among different races could help improve surgical outcomes. Moreover, breast MRI can capture aspects of breast tissue composition not reflected by density (14). Therefore, combining high-resolution DCE-MRI with molecular subtype, histology, recurrence, and survival outcomes specifically stratified by race remains an emerging area with limited literature. This study does not evaluate MRI−derived imaging biomarkers as predictors of outcome. Instead, the MRI−based cohort offers a longitudinal, well−characterized clinical population assembled through a standardized diagnostic pathway, allowing assessment of racial differences in molecular subtype distribution, recurrence patterns, and survival outcomes within a consistently defined cohort.

From a prognostic and recurrence point of view, prior studies showed that MRI is associated with smaller tumor size at recurrence, suggesting a potential survival benefit from earlier detection (15). By restricting the cohort exclusively on patients who received MRI, we aim to determine whether race itself, rather than disparity in access to MRI, is associated with risk of recurrence. Given that Black women experience higher rates of TNBC and lower rates of favorable hormone receptor (HR)-positive and human epidermal growth factor receptor 2 (HER2)-negative tumors, and that comprehensive MRI may improve tumor detection, we aimed to evaluate whether racial disparities in recurrence and survival persist within an MRI-ascertained cohort (16). This study aims to analyze data from the Duke-Breast-Cancer-MRI dataset (12), a longitudinal resource containing dynamic contrast-enhanced magnetic resonance imaging (DCE-MRI) and clinical information from breast cancer patients with known biopsy-confirmed tumor locations. Specifically, this study investigates differences in breast cancer subtypes, histology, recurrence rates, and survival outcomes among women of different racial backgrounds in a single consistently defined cohort. Using an MRI−ascertained population provides a standardized diagnostic context for evaluating race−associated differences in recurrence, survival and subtype distributions. This design moves beyond descriptive epidemiology by linking molecular subtype and longitudinal outcome data in the same patients, offering a clinically grounded exploratory framework for understanding racial disparities in prognosis, which remains underexplored in the existing literature.

## 2 Materials and methods

### 2.1 Study design and data source

This retrospective cohort study utilized the publicly available Duke-Breast-Cancer-MRI dataset (12), titled Dynamic Contrast-Enhanced Magnetic Resonance Images of Breast Cancer Patients with Tumor Locations, available through The Cancer Imaging Archive [https://www.cancerimagingarchive.net/collection/duke-breast-cancer-mri/]. All data were fully de-identified by the original curators in compliance with HIPAA guidelines. Institutional Review Board (IRB) approval was not required from our institution for this analysis, as the data are de-identified and publicly accessible.

The dataset includes records for 922 women diagnosed with biopsy-confirmed invasive breast cancer, all of whom underwent preoperative dynamic contrast-enhanced magnetic resonance imaging (DCE-MRI) at Duke University Hospital between January 1, 2000, and March 23, 2014. Inclusion criteria for this study were: histologically confirmed invasive breast carcinoma, availability of preoperative MRI data, and availability of clinical data relevant to tumor molecular subtype, recurrence status, and survival outcomes. Cases with key outcome variables were excluded from specific analyses as appropriate but retained in the overall cohort where feasible to maximize sample size.

### 2.2 Data extraction

The following variables were extracted and analyzed:

- Demographic Data and Race Grouping Strategy: Demographic data was based on patients’ age, gender, and race/ethnicity. For primary comparative analyses, race was categorized into three groups: White, Black, and Other. The “Other” category included Asian, Hispanic, Native, Hawaiian, American Indian, Multi-racial, and not recorded classifications. This grouping approach was selected *a priori* to improve statistical stability and interpretability given the small sample sizes in several individual racial strata. Secondary descriptive analyses retained full racial categories where appropriate.
- Tumor Characteristics: Histologic subtype, molecular classification (hormone receptor-positive [HR+], human epidermal growth factor receptor 2-positive [HER2+], TNBC), and nodal stage at diagnosis (number of involved lymph nodes). Two particularly important clinical categories include HR^+^ tumors, characterized by estrogen receptor (ER) and/or progesterone receptor (PR) positivity without HER2 overexpression, and TNBC which is defined by the absence of ER, PR, and HER2 expression.
- Imaging Data: MRI-derived tumor metrics and imaging-confirmed recurrence information.
- Survival and Recurrence: Time from diagnosis to recurrence, death, and last known-alive or last recurrence-free assessment from date of diagnosis.

All patients were followed from the date of diagnosis to the earliest occurrence of documented local or distant recurrence, death, or last documented recurrence-free clinical assessment. Recurrence was defined as the first occurrence of either local or distant breast cancer relapse. For patients without recurrence, the censoring time was defined as the later of the recorded date of death or last recurrence-free assessment. Death in the absence of recurrence was treated as a censoring event.

Histologic type was missing for 29.9% of patients and was addressed via multiple imputation by chained equations (MICE) in R (mice v3.14.0). Under a missing-at-random assumption, we included age at diagnosis, race/ethnicity, tumor grade and stage, ER/PR/HER2 status, and recurrence outcome as imputation predictors. Histologic categories (ductal, lobular, mixed, other) were imputed using polytomous logistic regression; continuous and binary variables employed predictive mean matching and logistic regression, respectively. We generated 20 imputed datasets with 10 iterations each, confirmed convergence by inspecting trace plots, and pooled descriptive and regression results across imputations using Rubin’s rules. Sensitivity analyses comparing imputed versus complete-case estimates showed negligible differences in histologic distributions and effect sizes, supporting the robustness of our findings.

Breast cancer subtypes were defined from immunohistochemistry (ER, PR, HER2) using the molecular-subtype code in Column G in the original dataset. For the statistical analyses we defined HR^+^ as any ER and/or PR-positive tumor regardless of HER2 status (molecular-subtype codes 0 or 1) and TNBC as ER-, PR-, and HER2-negative tumors (molecular-subtype code 3). All percentages used the number of patients in each racial group (mol-subtype available for the cohort) as the denominator unless otherwise stated.

### 2.3 Statistical analysis

All statistical analyses were performed using validated Python-based data science libraries. Descriptive statistics were used to summarize demographic and clinical characteristics. Categorical variables were expressed as counts and percentages; continuous variables were reported as medians with interquartile ranges (IQRs). Group comparisons were conducted using chi-square tests for categorical variables and Kruskal-Wallis or Mann-Whitney U tests for non-parametric continuous variables, as appropriate. For baseline and univariate analyses, comparisons were conducted across the three predefined racial groups (White, Black, and Other). Primary inferential comparisons focused on White versus Black patients, given their representation as the two largest racial groups in the cohort. Overall survival was analyzed using Kaplan–Meier methods, with between-group comparisons assessed using the log-rank test. Multivariable Cox proportional hazards regression was used to estimate adjusted hazard ratios (HRs) and 95% confidence intervals (CIs). Covariates included demographic and clinicopathologic factors at presentation, including age, menopausal status, histologic subtype, metastatic presentation, and Nottingham grade. Proportional hazards assumptions were evaluated using Schoenfeld residuals. Logistic regression was used to estimate unadjusted odds ratios (ORs) for triple-negative breast cancer (TNBC) by race. Given the limited number of mortality events, multivariable survival analyses were restricted to Black versus White comparisons to reduce model instability and overparameterization. All statistical tests were two-sided. A p-value < 0.05 was considered statistically significant throughout.

## 3 Results

### 3.1 Data preparation and descriptive statistics

A total of 922 women with biopsy-confirmed invasive breast carcinoma underwent preoperative magnetic resonance imaging (MRI). Ethnicity was reported for all 922 patients. Baseline demographic and clinical characteristics stratified by race (White, Black, and Other) are presented along with demographic variables in **Table 1**. The majority of participants were White (n= 651; 70.6%), followed by Black (n= 203; 22.0%); while other racial groups comprised only 68 (7.4%) patients.

**Table 1:** Baseline demographics and clinical characteristics of the cohort stratified by race.

Variable	Overall (n= 922)	White (n= 651)	Black (n= 203)	Other (n= 68)	p-value^*^
Age at diagnosis, years (median, IQR)	52.2 (45.4- 60.8)	53.3 (45.9- 61.8)	50.5 (44.0- 58.4)	49.2 (41.5- 54.9)	<0.001
Molecular subtype					
HR^+^	699 (75.8%)	517 (79.4%)	128 (63.1%)	54 (79.4%)	<0.001
TNBC	164 (17.8%)	98 (15.1%)	60 (29.6%)	6 (8.8%)	
Other/Unclassified	59 (6.4%)	36 (5.5%)	15 (7.4%)	8 (11.8%)	
Nodal stage at diagnosis^**^					
N0	529/898 (58.9%)	403/636 (63.4%)	94/197 (47.7%)	32/65 (49.2%)	<0.001
1–3 positive nodes	369/898 (41.1%)	233/636 (36.6%)	103/197 (52.3%)	33/65 (50.8%)	
Missing	24 (2.6% among all 922 participants)	15 (1.6% among all 922 participants)	6 (0.65% among all 922 participants)	3 (0.33% among all 922 participants)	
Histologic subtype^***^					
Ductal	575 (62.4%)	400 (61.4%)	127 (62.6%)	48 (70.6%)	0.135
Lobular	63 (6.8%)	52 (8.0%)	10 (4.9%)	1 (1.5%)	
Other histology	8 (0.9%)	7 (1.1%)	1 (0.5%)	0 (0.0%)	
Missing	276 (29.9%)	192 (29.5%)	65 (32.0%)	19 (27.9%)	
Nottingham Grade					
I	113 (17.7%)	92 (20.3%)	14 (10.1%)	7 (15.2%)	<0.001
II	318 (49.8%)	233 (51.3%)	59 (42.8%)	26 (56.5%)	
III	207 (32.4%)	129 (28.4%)	65 (47.1%)	13 (28.3%)	
Recurrence status					
Yes	87 (9.4%)	53 (8.1%)	27 (13.3%)	7 (10.3%)	0.090
No	833 (90.3%)	596 (91.6%)	176 (86.7%)	61 (89.7%)	
Missing	2 (0.2%)	2 (0.3%)	0 (0.0%)	0 (0.0%)	
Mortality (death recorded)					
Death	62 (6.7%)	36 (5.5%)	22 (10.8%)	4 (5.9%)	0.08
Alive/censored	860 (93.3%)	615 (94.5%)	181 (89.2%)	64 (94.1%)	

*p-values compare White *vs* Black *vs* Other (Kruskal-Wallis for age; chi-square for categorical variables).

**Nodal stage available for 898 patients (24 missing). Percentages for N0 and 1–3 are calculated among non-missing nodal stage.

***Histology comparison p-value calculated among non-missing histology only (ductal/lobular/other); missingness shown as a separate row.

Recurrence status was nearly complete (87 events, 833 non-events); 2 patients (0.2%) had missing recurrence status and were excluded from recurrence-specific analyses. ER, PR and HER2 marker data and molecular-subtype codes (0–3) were available for the full cohort. Histologic type was missing for 29.9% of the cohort (276/922); recorded-histology percentages therefore included the subset with non-missing histologic data (n = 646). Consequently, histologic type was addressed through multiple imputation in sensitivity analyses.

The median age at diagnosis (in years) was 52.2 (IQR 45.4-60.8). Age distributions differed significantly (p< 0.001) across the predefined racial groups (White, Black, and Other), with Black patients presenting at younger median ages compared with White patients **[****Table 1]**. There was no significant differences in the side (laterality) and menopausal status upon presentation.

When investigating Nottingham grade at presentation, 65 (47.1%) of black patients presented with stage III (P< 0.001). Overall, 29 (3.1%) of patients had metastatic presentation, with no significant differences between groups (p= 0.078). Molecular subtype distributions varied significantly between Black and White patients (p < 0.001). In particular, TNBC prevalence was higher among Black patients (60/203; 29.6%) than among White patients (15.1%). Molecular-subtype comparisons used the cohort of patients with complete ER/PR/HER2 results (molecular markers were available for the full worksheet) **[****Table 1]**. Nodal stage distributions are presented in **Table 1**. Nodal stage was not included in regression analyses unless explicitly stated.

### 3.2 Breast cancer subtype comparison by race

#### 3.2.1 Immunohistochemical (molecular) subtype distribution by race

Molecular subtype distributions differed significantly by race (p < 0.001), Black patients demonstrated higher prevalence of TNBC (29.6%) compared with White patients (15.1%), whereas HR^+^ tumors predominated among White patients **[****Table 1]**.

#### 3.2.2 Unadjusted odds of TNBC

In unadjusted regression analysis comparing Black and White patients, Black race was associated with significantly higher odds of TNBC compared with White race (OR = 2.47; 95% CI: 1.70-3.60; p < 0.001).

#### 3.2.3 Histological subtype distribution by race

Histological subtype information was available for 646 patients (70.1% of the cohort). Among patients with recorded histology, ductal carcinoma accounted for 89.0% of tumors, followed by lobular carcinoma (9.8%) and other histologies (1.2%). 276 records (29.9% of the full cohort) had missing histologic type. Comparison of histologic subtype (ductal, lobular, other) across the predefined racial groups did not demonstrate a statistically significant association (p= 0.135).

Examination of recorded histology (excluding missing entries) showed that ductal carcinoma predominated across all racial groups; among patients with recorded histologic type, ductal carcinoma accounted for 87.1% of recorded histologies in White patients, and 92.2% in Black patients. Lobular carcinoma was more common among White patients (11.3%) than Black patients (4.9%) **[****Table 1]**.

The comparison of the three histologic categories (ductal, lobular, other) across the predefined racial groups did not reveal a statistically significant association.

### 3.3 Recurrence

Recurrence proportions stratified by race are shown in **Table 1**. Black patients demonstrated a higher crude recurrence proportion compared with White patients (13.3% *vs* 8.1%); however, this difference did not reach statistical significance in unadjusted comparisons (p = 0.090). No time-to-event modeling for recurrence was performed.

### 3.4 Survival analysis

Overall survival time was defined as days from diagnosis to death when applicable; otherwise, patients were censored at the last-known-alive follow-up time. Patients lacking both death time and follow-up time were excluded from survival time-to-event analyses. Median follow-up for the cohort (event time for decedents; otherwise censoring time) was 1,415 days (IQR 835-1,805 days).

**Figure 1:**
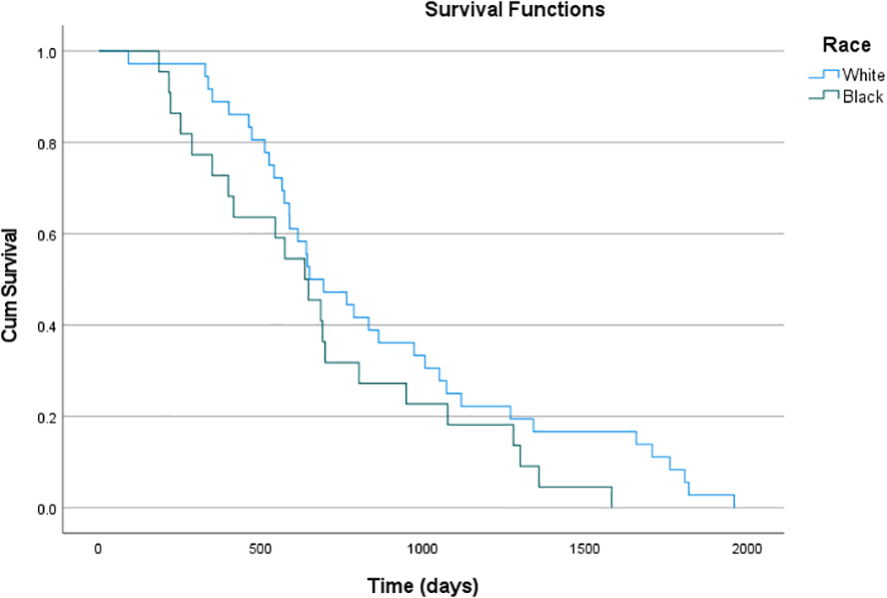
Kaplan-Meier curves for overall survival (in days) by race plotted against the cumulative survival beyond the given time-points.

Because the majority of patients were censored for mortality (fewer than half of patients experienced the event), a median overall survival was not estimable. Kaplan-Meier survival curves stratified by race are shown in **Figure 1**. Kaplan-Meier survival curves demonstrated lower overall survival among Black patients compared with White patients. In pairwise log-rank testing, survival differed significantly between Black and White patients (p= 0.009). No additional race-specific survival comparisons were performed due to limited sample sizes in smaller subgroups. The mortality rate for Black patients was 10.8%, compared to 5.5% among White patients, with an overall mortality rate of 6.7% in this cohort (p= 0.08).

Multivariable Cox proportional hazards models were used incorporating demographic and clinicopathologic factors at presentation irrespective of univariable statistical significance. Covariates included age at diagnosis, menopausal status, histologic subtype, metastatic presentation, and Nottingham grade, with stratification by race. the overall likelihood ratio test did not demonstrate a statistically significant improvement in model fit compared with the null model (χ² = 6.605, df = 6, p = 0.359). Harrell’s concordance index indicated limited discriminative performance. None of the included covariates was independently associated with mortality after multivariable adjustment.

Given the modest number of observed death events relative to the number of covariates included, these multivariable estimates should be interpreted cautiously and are presented as exploratory, as these findings describe associations observed in the available data and do not establish causal effects.

### 3.5 Association between prognosis and cancer type

Analysis of histological subtype showed that invasive ductal carcinoma (IDC) was associated with significantly better survival compared to invasive lobular carcinoma (ILC). The median survival for IDC was 1,282 days versus 1,025 days for ILC (p = 0.003, log-rank test). In terms of recurrence among patients with recorded histology and non-missing recurrence status, IDC had a recurrence proportion of 9.0% (52/575). while ILC had a recurrence proportion of 12.7% (8/63). This difference was not statistically significant (χ² = 0.51, p = 0.474). Comparisons of prognosis by histologic subtype were limited by missing histology and outcome data. Given the modest number of recurrence and death events, these findings should be interpreted cautiously and were not intended to support definitive comparative inference.

### 3.6 Summary of findings

In summary, baseline characteristics differed by race in this MRI-ascertained cohort. Age at diagnosis and nodal stage distribution varied significantly across the primary racial groups. Molecular subtype distributions differed significantly, driven by a higher prevalence of TNBC among Black patients. In comparison, histologic subtype distributions among patients with recorded histology did not significantly differ by race, and ductal carcinoma predominated across all groups. Crude recurrence proportions were higher among Black patients than White patients, although this difference did not reach statistical significance in unadjusted comparisons. The mortality rate followed the same trend, with Kaplan-Meier survival curves demonstrating non-statistically significant lower overall survival among Black patients.

## 4 Discussion

This study builds upon existing evidence regarding racial disparities in breast cancer, highlighting significant differences in molecular subtype distribution, recurrence patterns, and survival outcomes across racially diverse groups. Most notably, Black women in our cohort exhibited the highest recurrence rates and significantly shorter survival durations compared to other groups, reinforcing their persistent vulnerability to poorer breast cancer outcomes (2-5).

Analysis of molecular subtypes revealed a disproportionately higher occurrence of TNBC among Black women, which is a subtype known for its aggressive clinical course and limited treatment options. In contrast, HR+ tumors predominated among White patients. The distribution of these molecular subtypes helps contextualize the observed differences in outcomes of breast malignancies among different racial groups and reinforces the relevance of stratifying breast cancer data by race (17).

Histologic subtype analysis showed that IDC was the dominant form across all groups, with no statistically significant variation by race. Contrary to earlier assumptions, ILC did not disproportionately affect any particular racial group in our verified data. Furthermore, while Hispanic women were previously thought to show unique subtype patterns, our recalculated findings suggest a more homogeneous IDC distribution across racial groups.

The current data suggested lower overall survival among Black compared with White patients. These findings should be interpreted cautiously and are best viewed as hypothesis-generating exploratory analysis rather than confirmatory. Notably, in multivariable Cox regression adjusting for histologic subtype and age, Black race remained associated with a higher hazard of death, suggesting that observed survival differences are not fully explained by tumor histology alone. Given the limited number of mortality events and substantial censoring, these associations should be interpreted carefully, keeping into consideration systemic factors beyond measured tumor characteristics, such as care environments, healthcare access, and treatment delivery may contribute to outcome disparities (18, 19).

Biological and systemic mechanisms likely contribute to observed disparities. Higher prevalence of aggressive subtypes such as TNBC has been documented among Black women, and tend to have higher rates of tumor-associated mutations, including TP53 and BRCA1/2 (20, 21). Yet, these biological explanations are insufficient on their own. Socioeconomic barriers, including limited access to screening, insurance limitations, diagnostic delays, and lower adherence to treatment, may exacerbate these disparities (22). Moreover, previous literature demonstrated that inequitable care environments can undermine outcomes regardless of biological disease characteristics (18, 19).

From a clinical standpoint, these findings support the urgent need for precision oncology frameworks tailored to high-risk populations. Integrating molecular diagnostics with advanced imaging techniques like multiparametric MRI and contrast-enhanced spectral mammography may help in improving diagnostic accuracy in underserved groups. Additionally, adopting culturally responsive risk assessment models, community engagement, and developing personalized surveillance plans are crucial to address recurrence and reduce mortality rates in specific marginalized populations.

Racial disparities in breast cancer subtype distribution and outcomes are well documented, including the higher prevalence of aggressive subtypes and poorer survival among Black women (6). The present study extends this literature by providing an integrated, longitudinal assessment of subtype, recurrence patterns and overall survival within a consistently defined cohort. Utilizing an MRI−ascertained dataset with standardized clinical annotation and follow−up minimizes heterogeneity common to multi−source registries and enables assessment of disparities across multiple points in the disease course rather than examining subtype or survival in isolation. This framework also establishes a clinically grounded platform for future investigations aimed at determining whether quantitative imaging biomarkers offer prognostic value beyond established clinical and pathologic factors (12).

This study has several limitations. As a retrospective cohort analysis, there are constraints in reliably determining cause-and-effect relationships, and unmeasured confounding factors may exist. Missing data in histologic, recurrence, and survival fields may introduce selection bias despite sensitivity analysis. Key variables, including comorbidities, treatment regimens, and sociodemographic factors such as income, education, insurance status, and geographic access to care were unavailable, potentially influencing observed associations. While subtype classification relied on standard histopathology and immunohistochemistry, deeper genomic profiling was not available, limiting exploration of underlying molecular mechanisms.

Although derived from a breast MRI dataset, our analyses focused on clinical and pathologic variables and did not incorporate quantitative MRI features such as enhancement kinetics, lesion morphology, or radiomic signatures. As a result, the current findings should be interpreted as characterizing disparities in subtype, recurrence, and survival within an MRI-ascertained cohort rather than attributing observed differences to imaging phenotypes. This framework provides a foundation for future studies evaluating whether imaging biomarkers offer incremental prognostic value beyond standard clinical factors.

A key limitation of the survival analysis is the modest number of observed death events relative to the number of covariates considered, resulting in limited event-per-variable ratios that reduced statistical power and constrain the discriminative performance of multivariable Cox regression estimates. Although this reflects favorable overall survival within this cohort but further limits the ability to detect independent prognostic associations.

Observed racial differences should not be interpreted as intrinsic to tumor biology alone. Extensive evidence across medicine demonstrates that structural inequities, including differential access to care, treatment delivery, and follow-up contribute to outcome disparities (23). This underscores the importance of interpreting race-associated outcomes within the broader healthcare context.

## 5 Conclusions

This study highlights persistent racial disparities in breast cancer survival within an MRI-ascertained longitudinal cohort, particularly among Black women who had highest prevalence of TNBC (29.6%) and stage III Nottingham grade at presentation 47.1%. The overall recurrence rate was 9.4%, and mortality rate was 6.7%, with no statistically significant difference between groups. While prior work has documented similar inequities, the present analysis offers an integrated assessment of molecular subtype, recurrence patterns, and survival within a consistently annotated dataset, enabling evaluation of disparities across the disease continuum. Given the observational design, these associations should be interpreted cautiously and cannot establish causality. These observed disparities likely reflect the combination of tumor biology and socioeconomic inequities, which are prevalent throughout medicine. To improve equity, oncology frameworks must go beyond biology, incorporating culturally responsive screening, equitable access to care, and race-conscious follow-up strategies. Future prospective, multi-institutional research should integrate genomic, imaging, and socioeconomic data to comprehensively examine the interaction of biological mechanisms and structural determinants to guide precision interventions aimed at reducing outcome disparities.

## Data Availability

Publicly available datasets were analyzed in this study. This data can be found here: The Duke-Breast-Cancer-MRI dataset, which is available online under the title “Dynamic Contrast-Enhanced Magnetic Resonance Images of Breast Cancer Patients with Tumor Locations”, available through The Cancer Imaging Archive [https://www.cancerimagingarchive.net/collection/duke-breast-cancer-mri/].
